# Zero-valent Fe confined mesoporous silica nanocarriers (Fe(0) @ MCM-41) for targeting experimental orthotopic glioma in rats

**DOI:** 10.1038/srep29247

**Published:** 2016-07-08

**Authors:** M. A. Shevtsov, M. A. Parr, V. A. Ryzhov, E. G. Zemtsova, A. Yu Arbenin, A. N. Ponomareva, V. M. Smirnov, G. Multhoff

**Affiliations:** 1 Klinikum rechts der Isar, Department Radiation Oncology, Technische Universität München, Ismaniger Str. 22, Munich 81675, Germany; 2 Institute of Cytology of the Russian Academy of Sciences (RAS), St. Petersburg, 194064 Tikhoretsky ave., 4, Russia; 3 Saint Petersburg State University, St. Petersburg, Universitetskaya nab. 7 - 9, 199034, Russia; 4 NRC “Kurchatov Institute”, B.P. Konstantinov Petersburg Nuclear Physics Institute, Gatchina 188300, Russia

## Abstract

Mesoporous silica nanoparticles (MSNs) impregnated with zero-valent Fe (Fe(0) @ MCM-41) represent an attractive nanocarrier system for drug delivery into tumor cells. The major goal of this work was to assess whether MSNs can penetrate the blood-brain barrier in a glioblastoma rat model. Synthesized MSNs nanomaterials were characterized by energy dispersive X-ray spectroscopy, measurements of X-ray diffraction, scanning electron microscopy and Mössbauer spectroscopy. For the detection of the MSNs by MR and for biodistribution studies MSNs were labeled with zero-valent Fe. Subsequent magnetometry and nonlinear-longitudinal-response-*M*_*2*_ (NLR-*M*_*2*_) measurements confirmed the MR negative contrast enhancement properties of the nanoparticles. After incubation of different tumor (C6 glioma, U87 glioma, K562 erythroleukemia, HeLa cervix carcinoma) and normal cells such as fibroblasts and peripheral blood mononuclear cells (PBMCs) MSNs rapidly get internalized into the cytosol. Intracellular residing MSNs result in an enhanced cytotoxicity as Fe(0) @ MCM-41 promote the reactive oxygen species production. MRI and histological studies indicated an accumulation of intravenously injected Fe(0) @ MCM-41 MSNs in orthotopic C6 glioma model. Biodistribution studies with measurements of second harmonic of magnetization demonstrated an increased and dose-dependent retention of MSNs in tumor tissues. Taken together, this study demonstrates that MSNs can enter the blood-brain barrier and accumulate in tumorous tissues.

Glioblastoma multiforme (GBM) is highly aggressive primary brain tumor that is associated with high morbidity and poor prognosis. Despite multimodality treatment approach that currently includes maximal surgical resection, followed by a combination of radiotherapy and/or chemotherapy with temozolomide (TMZ), the median survival does not exceed 15 months^1^. Targetable nanocarriers for treating malignant gliomas are a unique way to overcome chemotherapeutic levels at target sites devoid of systemic toxicity^2^. One of the currently available nanocarrier systems are nanoparticles based on silica.

Mesoporous silica-based nanoparticles (MSNs) represent an attractive system for a controlled release of anticancer drugs including chemotherapeutic agents, small interfering RNAs (siRNA), radiotherapy sensitizers^3^,^4^,^5^,^6^. Numerous studies showed the possibility of efficient drug delivery by MSNs^7^,^8^,^9^,^10^,^11^. Thus great immune-stimulating activity *in vivo* was provided by MSNs, which carried oligodeoxynucleotides (ODN) containing cytosine-guanine (CpG), in comparison to the control free CpG therapeutics, as it was found in the study by Zheng *et al*.^12^. For further tumor-specific targeting MSNs could be functionalized with various bioligands^13^,^14^,^15^. In the study by Goel *et al*. MSNs coated with anti-VEGFR ligand VEGF121 could deliver significantly higher amounts of sunitinib to U87MG when compared with the non-targeted counterparts^14^. In another study by He *et al*. it was shown that MSNs coated with seleno-amino acid and conjugated with TAT cell penetrating peptide and transferrin (SeC @ MSNs-Tf/TAT) preferentially accumulated in cancer cells due to the receptor-mediated endocytosis^15^.

Up-to-date studies demonstrated the therapeutic efficacy of MSNs for targeting glioma cells in *in vitro* experiments. However, the question whether nanoparticles can penetrate the brain-tumor barrier still remained to be elucidated. In a recent work of Huang *et al*. retention of MSNs was revealed in an orthotopic U87MG glioblastoma model where mesenchymal stem cells were applied for the transportation of the MSNs^16^. In the present investigations the ability of Fe(0) @ MCM-41 nanoparticles for targeting the brain tumor was investigated in the model of orthotopic C6 glioma with application of the magnetic resonance imaging (MRI). Moreover, the study of biodistribution of magnetic nanoparticles was carried out applying the sensitive measurements of second harmonic generation in parallel *dc H* and weak *ac h(t)* magnetic fields (NLR-*M*_*2*_)^17^.

## Results

### Physico-chemical characterization of Fe(0) @ MCM-41 nanoparticles

**Figure 1:**
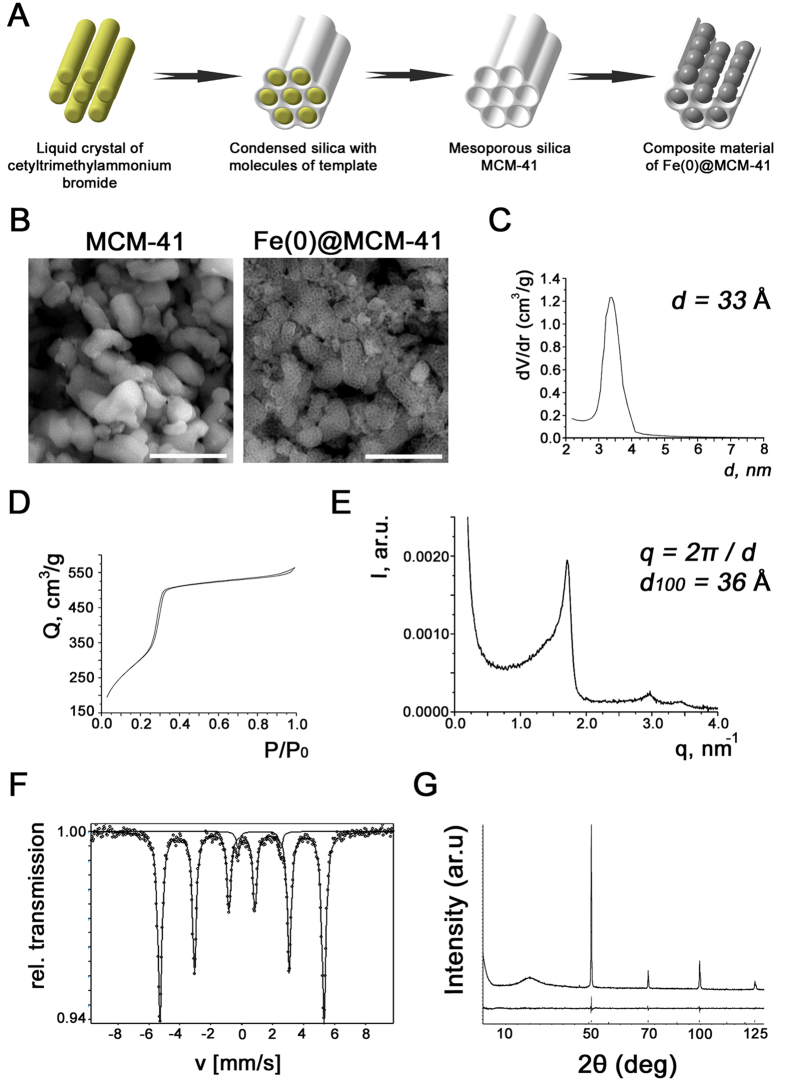
Synthesis and characterization of the Fe(0) @ MCM-41 nanoparticles. (**A**) Schematic representation of Fe(0) @ MCM-41 synthesis. (**B**) SEM images of the MSNs and zero-valent Fe impregnated particles. Scale bar, 100 nm. **(C)** Differrential distribution curve of pores in the Fe(0) @ MCM-41 particles. **(D)** Nitrogen isotherm of the calcinated MSNs sample. (**E**) Energy dispersive X-ray (EDX) spectra of Fe(0) @ MCM-41. **(F)** Mössbauer spectra of Fe-MSNs particles. **(G**) X-ray difractometry pattern of Fe(0) @ MCM-41 nanoparticles.

**Figure 2:**
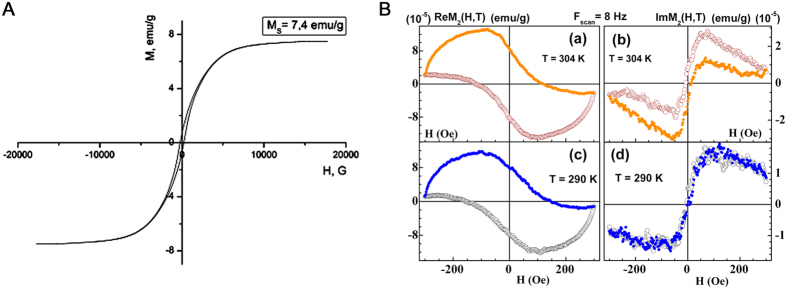
Magnetic measurements of zero-valent Fe containing MSNs. (**A**) Magnetization hysteresis loop of Fe(0) @ MCM-41 nanoparticles. **(B)** Phase components of second harmonic magnetic response of Fe(0) @ MCM-41 solution (C≈0.01 mg/ml) to a weak *ac* field.

The morphology of the synthesized MSNs was determined by means of scanning electron microscopy (SEM) (Fig. 1). Figure 1B demonstrates clearly the porous structure of the nanoparticles. Their average length constituted 250 nm and the average diameters varied from 100 to 150 nm. The average diameter of pores constituted 3.3 nm (Fig. 1C). Figure 1D shows the nitrogen adsorption isotherm of the calcinated Fe(0) @ MCM-41 particles, clearly indicating the increase of the nitrogen absorption with the increase of the pressure. Following incorporation of the zero-valent Fe into the MSNs we could detect the ferrum in the pores though we did not observe the change in the size of the particles. Comparison of spectra of energy dispersive X-ray (EDX) from Fe(0) @ MCM-41 nanoparticles and empty MSNs demonstrated the presence of the peak originating from iron (Fig. 1E). X-ray difractometry registered with a time of data collection of 2 s pre each step and angle step of 0.01° reveals the crystalline α-Fe phase in the Fe(0) @ MCM-41 nanoparticles (Fig. 1G). The presence of zero-valent Fe in samples with the MNSs was supported by subsequent Mössbauer measurements (Fig. 1F). Figure 2A presents the magnetization hysteresis of Fe(0) @ MCM-41 which has been obtained in steady field varied within –1 T – 1 T at room temperature. Saturation magnetization (*M*_*s*_) constituted 7.4 emu/g. To analyze the magnetic state of Fe(0) @ MCM-41 particles the NLR setup was applied, registering the magnetic response on second harmonic, *M*_2_, (Fig. 2B). Both phase components, Re*M*_2_ and Im*M*_2_ were recorded simultaneously in dependence on steady field *H* at varied frequency of field scan, *F*_sc_, and constant *T* = 294 K under condition *M*_2_ ∝ *h*^2^ (*h* – is amplitude of *ac* field) (Supplementary Fig. S1). The concentration of Fe(0) @ MCM-41 was about 0.01 mg/ml. The presented signals reveal extremes in a weak *H* ~100 Oe for both phase components Re*M*_2_, Im*M*_2_, exhibiting inverse signs, and discrepancy in their *H-*dependencies registered during direct and reverse scannings of steady field *H* (field hysteresis). The latter decreases with decreasing of *F*_sc_, evidencing its dynamical reason. The listed peculiarities of *M*_2_-response are characteristic for that of ensemble of magnetic nanoparticles in single domain regime, which do not interact with each other, near blocking temperature like these of other similar ensembles^17^. The converse signs of obtained signals in real Re*M*_2_ and imaginary Im*M*_2_ components suggest respectively the distinct major reasons of their origination: (i) a nonlinear dependence on field of the magnetization of MSN ensemble, *M*, in Re*M*_2_ ∝ ∂^2^*M/∂H*^2^; and (ii) an external field influence on relaxation processes in Im*M*_2_ ∝ (∂Γ/∂*H*)·(∂*M/∂H*) (here Γ = 1/*τ* is the magnetic relaxation rate). These findings indicated that interaction of Fe(0)-nanoparticles intercalated inside the porous Fe(0) @ MCM-41 composite particle is rather weak and they did not form multidomain magnetic system inside single particle.

### Cellular interactions of mesoporous silica nanoparticles

**Figure 3:**
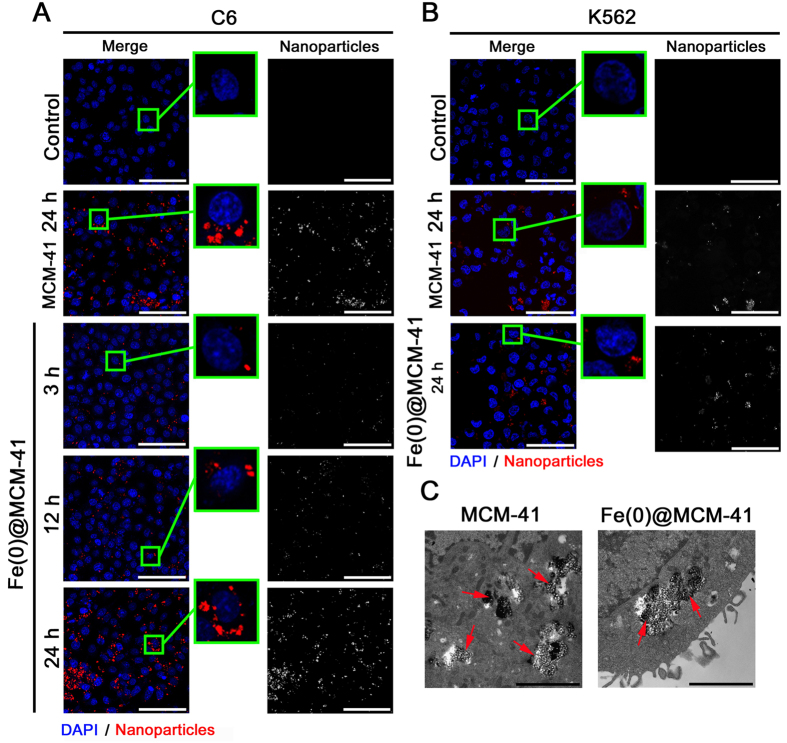
Cellular interactions of MSNs. **(A)** Immunofluorescent images of C6 cells following 24 hours incubation with PBS, MSNs and Fe-MSNs. Nuclei were stained with DAPI (blue). Nanoparticles were detected by reflecting laser scanning at 488 nm (red). Scale bar, 10 μm. (**B**) Immunofluorescent images of K562 cells following 24 hours of incubation with PBS, MCM-41 and Fe(0) @ MCM-41 particles. **(C)** Transmission electron microscopy of the C6 cells co-incubated with MSNs and Fe(0) @ MCM-41 particles. Scale bar, 200 nm.

**Figure 4:**
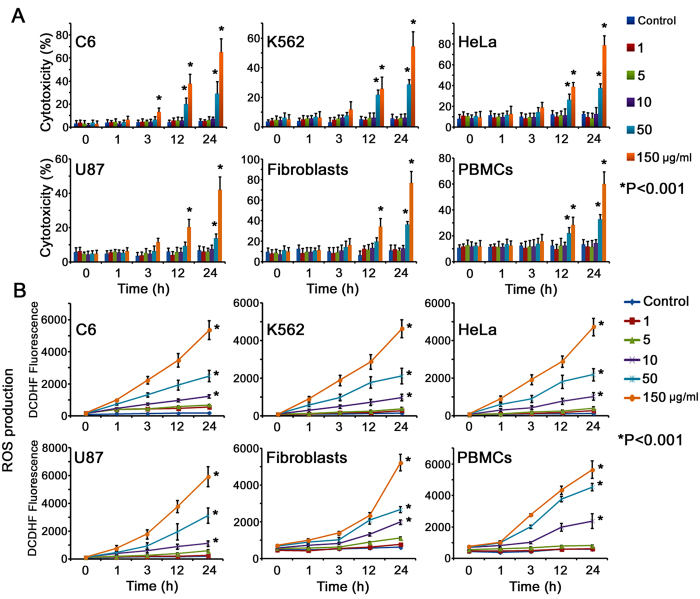
Cytotoxicity analysis of the Fe(0) @ MCM-41 particles. **(A)** Cytotoxicity assay of the tumor and normal cells incubated with particles. Each value represents mean ± S.D. from three independent experiments. **(B)** Kinetics of ROS production following cells exposure to MSNs and Fe-MSNs. Each value represents mean ± S.D. from four independent experiments.

Internalization and cytotoxicity of the developed MCM-41 and Fe(0) @ MCM-41 particles were assessed in rat C6 glioma, human U87 glioblastoma, human leukemia K562 and cervix carcinoma HeLa cells. Rat’s splenocytes and fibroblasts were used as normal tissue cells. Cells were co-incubated for 1, 3, 6, 12, and 24 hours with Fe(0) @ MCM-41 nanoparticles at different concentrations of Fe (C = 1, 10, 50, 150 μg/ml). Analysis of confocal microscopy data clearly indicated the accumulation of Fe(0) @ MCM-41 as well as MCM-41 particles in cytosol of cancer cells as a result of overnight co-incubation (Fig. 3). Indeed, the particles can be seen as surrounding the nucleus as red dots in all cell types on the confocal microscopy images obtained in reflective regime (Fig. 3A). The highest incorporation of the nanoparticles was observed 24 hours after co-incubation. The similar dynamics of particles incorporation was also observed when fibroblasts or PBMCs were applied. Subsequent transmission electron microscopy (TEM) of C6 cells indicated the existence of the dense structures in the cytoplasm, well scattering the electrons, which corresponds to MCM-41 and Fe(0) @ MCM-41 (Fig. 3C). Cytotoxicity assay demonstrated that Fe(0) @ MCM-41 but not MCM-41 nanoparticles did exhibit cytotoxicity towards tumor cells and normal cells (Fig. 4). Thus 12 hours following cell exposure to Fe(0) @ MCM-41 particles (at 150 μg/ml) there was an increase of cytotoxicity up to 40% (Fig. 4A). The cytotoxicity was further increased at time point of 24 hours which corresponded to the highest incorporation of particles (confirmed by confocal microscopy). An involvement of reactive oxygen species (ROS) could be the cause for the elevated cell death. Therefore the rate of ROS production was gauged by us in tumor and normal cells both under normal growth conditions and after co-incubation with MCM-41 and Fe(0) @ MCM-41 nanoparticles. ROS were tested by applying a fluorescent probe of dichlorodihydrofluorescein diacetate (DCDHF) (Molecular Probes, USA). When MCM-41 particles were applied no considerable growth of ROS production was detected in all tested cells (*data not shown*). However, the registered ROS production rate was noticeably enhanced in the cells treated with Fe(0) @ MCM-41 nanoparticles (Fig. 4B). The increasing depended on the concentration of the Fe, time of co-incubation with nanocarriers as well as cell type.

### Fe(0) @ MCM-41 nanocarriers accumulate in glioma

**Figure 5:**
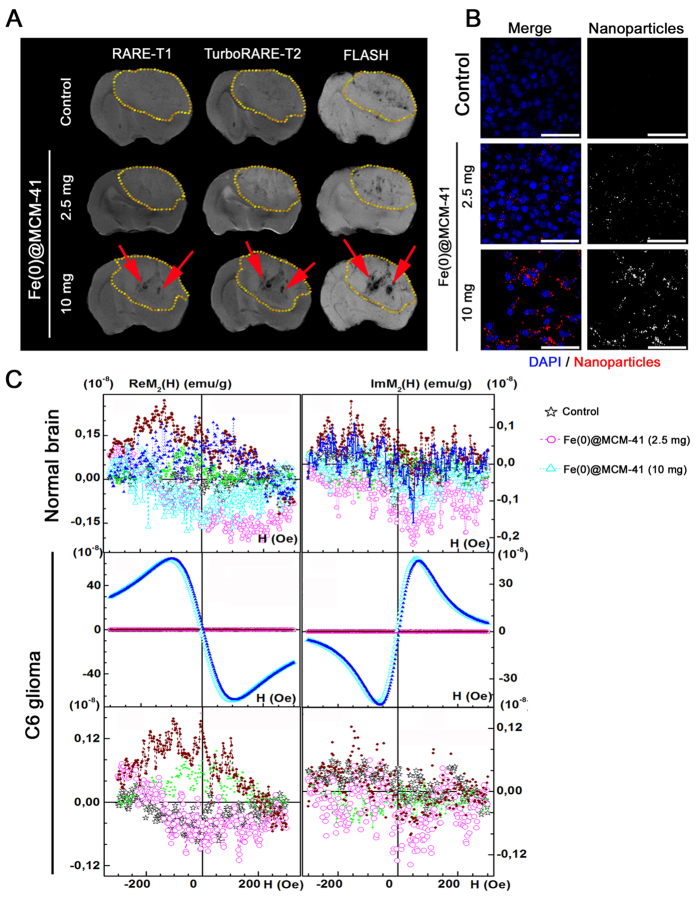
Accumulation of the Fe(0) @ MCM-41 nanoparticles in the glioma. (**A)** Magnetic resonance images for the control, animals treated with Fe(0) @ MCM-41 at dosage of 2.5 and 10 mg/kg. Images were obtained in RARE-T1, TurboRARE-T2 and FLASH regimens. Retention of the nanoparticles in the tumor presented as hypotensive zones on T2-weighted and gradient echo images (red arrows). **(B)** Immunofluorescent images of the brain tumors for the control and animals treated with Fe(0) @ MCM-41. Nuclei stained with DAPI (blue). Nanoparticles detected using reflective laser scanning at 488 nm (red). Scale bar, 40 μm. **(C)** Biodistribution of Fe-MSNs nanoparticles in normal brain and tumor of the treated animals from NLR- *M*_2_ data. Solid symbols are used for curves recorded at direct *H*-scan and open symbols for the reverse one.

On day 15 after intracranial injection of C6 cells a random arrangement of all animals was performed between four groups (6 animals in each) by the following way: (1) control group after phosphate-buffer solution (PBS) injection intravenously; (2) I.V. injection of MCM-41; (3) the Fe(0) @ MCM-41 injection intravenously by dose of 2.5 mg/kg during 24 hours; (4) I.V. injection of Fe(0) @ MCM-41 by dose of 10 mg/kg during 24 hours. After intravenous infusion of the particles no obvious side effects were observed (i.e., behavioral changes). Following intravenous administration of Fe(0) @ MCM-41 the MR images exhibiting high-resolution were registered (Fig. 5A). The C6 glioma being visible on MR images as hypotensive zone at registration of scans in regime the *T*_1_-weighting, became slightly hypertensive on those obtained in the *T*_2_-weighted regime. Infusion of the MCM-41 nanoparticles did not lead to the enhancement of the tumor contrast in MR scans. Following Fe(0) @ MCM-41 injection the origination in the glioma of the ‘dark’ hypotensive zones was found on the images registered in *T*_2_-weighted regimen. The latter became more contrast at application of the FLASH regimen (Fig. 5A). When conjugates were injected by the 10 mg/kg dose retention of the MSNs nanoparticles in the tumor was significantly higher compared to the lesser (2.5 mg/kg) dose. This enhanced the tumor contrast at applying scans with T2-weighting and Fe(0) @ MCM-41 nanoparticles could be detected in patchy pattern of hypotensive zones. Multigradient sequences with T2^*^-weighting (MSME regimen) have been additionally exploited to estimate *T*_2_* values. The determined quantity of *T*_2_* was 45 ± 6 s^−1^ in control group. Intravenous injection of Fe(0) @ MCM-41 resulted in a decrease of *T*_2_* magnitude to 32 ± 3 s^−1^, that was further dropped down to 21 ± 6 s^−1^ (P < 0.001) when 10 mg/kg dose was used. In the animals treated with MCM-41 without the Fe the T2* values did not significantly differ from that of control group constituting 46 ± 3 s^−1^. Subsequent immunofluorescence staining demonstrated an accumulation of the Fe(0) @ MCM-41 in the glioma tissue when dose of 2.5 mg/kg was used (Fig. 5B). Nanoparticles localized in the extracellular space in the perivascular zones in the tumor tissue. When particles were injected at dosage of 10 mg/kg we observed a significant increase of Fe(0) @ MCM-41 retention in the tumor.

### Biodistribution analyses of nanoparticles

Analysis of the Fe(0) @ MCM-41 nanoparticles accumulation in the tissue of intact brain of glioma-bearing rodents by NLR-*M*_*2*_ measurements did not indicate the retention of the conjgates in the brain tissue (Fig. 5C). These data suggest that non-magnetic MCM-41 particles do not penetrate also the intact blood-brain barrier. Incorporation of MCM-41 into glioma cells can be qualitatively assessed from *in vitro* experiments with C6 cells (confocal microscopy and TEM data, Fig. 3A,C). When the tumor samples were obtained from rats treated with dose 2.5 mg/kg of the Fe(0) @ MCM-41 the incorporation of the latter in tumor can be seen on the immunofluorescent images (Fig. 5B). However, the Re*M*_2_ response from tumor only slightly exceeded that from control untreated (or treated with MCM-41) animals (with ratio 1.12; Table 1) and exhibited considerable *H*-hysteresis (Fig. 5C) similar to this in signal of Fe(0) @ MCM-41 in suspension solution (Fig. 2). The data suggest that there is no release of Fe(0) from pores of silica particles followed by oxidation of Fe(0) to Fe_3_O_4_ and appearance of non-hysteretic *M*_2_(*H*)-signal like that for 10 mg/kg dosage (see below) (Fig. 5C; Table 1). Administration of Fe(0) @ MCM-41 at dosage of 10 mg/kg enhanced the accumulation of the MSNs in the glioma with subsequent release of Fe(0). Its oxidation was accompanied by increase of the non-hysteretic *M*_2_(*H*)-response (Fig. 5C), which was similar to that of Fe_3_O_4_-based nanoparticles used in earlier investigations^17^,^18^. The dramatic enhancement (~770-fold) of magnetic nanoparticle accumulation in glioma in comparison to 2.5 mg/kg dosage (Table 1) indicated the proportional increase of concentration of Fe(0) released from the MCM-41, since the amplitude of Re*M*_2_ ∝ NP’s concentration^17^. The effect of magnetic NPs on contrast of MR images occurred through changing of the relaxation characteristics *T*_1_, *T*_2_, *T*_2_^*^, which depended on the NPs distribution (Fig. 5A). Biodistribution of magnetic nanoparticles in tissues of treated rodents was assessed by study of nonlinear response in longitudinal geometry of parallel weak *ac*, *h*(*t*) = *h·*sin*ωt*, and *dc*, *H*, magnetic fields, second harmonic of magnetization, *M*_2_(*H*), being registered. Intriguingly, a weak *M*_2_(*H*) signal exhibiting field hysteresis and extremum in a small field *H* ~ 50 Oe was found in some tissues of control non-treated animals (Supplementary Fig. S2). Signals were detected in tissues of tumor, skin, heart and muscle. The latter implies the presence of some magnetic nanoparticles in certain intact tissues. Reasons for this still remain unclear; one may suggest that the latter are presumably involved in a cell metabolism. Control tissue’s *M*_2_ signals were always significantly weaker of those from corresponding tissues (including tumor) of MNP-treated animals^17^. In current study with use of Fe(0) @ MCM-41, an exception was detected only for heart tissue, where the amplitudes of these signals were comparable (Supplementary Fig. S2). Following injection of Fe(0) @ MCM-41 by dose 2.5 mg/kg the relative retention of MNP in tissues and organs increased as follows: tumor < muscle ≤ heart < brain < lungs < skin < kidney ≪ liver < spleen (Table 1). In 24 hours after administration of Fe(0) @  @ MCM-41 at dosage of 10 mg/kg the Re*M*_2_(*H*) response enhanced in tumor and in less extent in lungs, as well as the order of relative increase of MNP retention in tissues changed in the following way: muscle ≤ brain ≈ kidney ≈ heart < skin ≪ lungs < tumor < spleen < liver (Supplementary Fig. S3 and Table 1). The interesting peculiarity of obtained *M*_2_(*H*) dependences, which can be seen from this Figure, is their non-hysteretic character in responses from spleen, liver, tumor and slightly hysteretic one from lungs tissue, which corresponds to response of Fe_3_O_4_-based magnetic NPs in superparamagnetic regime employed in previous studies^17^ and differs drastically from hysteretic *M*_2_-signal of Fe(0) @ MCM-41 in suspension solution (Fig. 2). At the same time the other tissues reveal the rather weak hysteretic response corresponding to that of NP solution and comparable in value with control signals (Supplementary Fig. S1). This suggests that the Si-based shell of composite is destroyed in first group of tissues, as can be expected, releasing the Fe(0) NPs, and the latter is further oxidized to Fe_3_O_4_ = Fe_2_O_3_ × FeO state. In lungs, where content of oxygen is higher, the oxidation leads to some larger amount of Fe_2_O_3_ as compared to FeO, and we observed some *H-*hysteresis, since *M*_2_ response of the former is characterized by *H*-hysteresis. Thus the obtained results imply the possible Fe(0) oxidation in case of Fe(0) @ MCM-41 particles.

## Discussion

The application of mesoporous nanocarriers in the oncology field opens a promising perspective owing to the ability for transportation of therapeutic agents and their liberation in tumor in a controlled manner^19^. For the assessment of the biodistribution of MSNs in animal brain tumors Fe(0) was confined into the pores of the nanoparticles. The obtained nanocarriers had the properties of T2-negative contrast enhancers (Figs 1 and 2).

When we applied non-impregnated with Fe(0) MCM-41 nanoparticles we did not observe any cellular toxicity. The obtained results are in accordance with data published recently by Sadeghnia *et al*. showing that exposure of MSNs to rat pheochromocytoma PC12 cells (1.95–1000 μg/mL) did not result in reduction of cell viability. The amount of intracellular reactive oxygen species (ROS) and oxidative DNA damage also did not differ in these nanoparticles^20^. When Fe(0) was incorporated into the pores of the nanoparticles we observed a subsequent increase of cellular cytotoxicity (Fig. 4). This could be attributed to the oxidative stress induced by glutathione depletion, followed by ROS induction and peroxidation of lipids^21^. Previously in the work by Watanabe *et al*. it was shown that Fe_3_O_4_ magnetic nanoparticles (MgNPs- Fe_3_O_4_) provided the growth of ROS production even at lower concentration (10 μg/mL) followed by increase of oxidative damage of DNA, and reduction of the glutathione level; at high concentration (100 μg/mL) they caused cell membrane damage^22^. High ROS levels can subsequently damage cells by disrupting DNA, altering gene transcription that could result in activation of apoptotic pathway^23^,^24^,^25^. Increased amounts of ROS also might damage the mitochondrial DNA (mDNA)^26^. Furthermore, it was demonstrated that oxidative stress induced by nanoparticles affects cell signaling activating inflammation through NF-κB^27^. It was found also that Fe(0) @ MCM-41 nanoparticles reveal a cytotoxicity which depends on the dose. Similar results were reported previously for silica-coated iron oxide nanoparticles for the primary human macrophages, dendritic cells and A549 human lung adenocarcinoma cells^28^,^29^. Subsequent measurement of ROS production confirmed this mechanism in our data set (Fig. 4B). One of the possible solutions for reduction of Fe(0) @ MCM-41 toxicity could be the functionalization or coating of the MSNs surface with various ligands including proteins, sugar moieties, hyperbranched poly(ethylene imine), PEG^30^,^31^,^32^. Thus end-capping of MSNs with such natural proteins as bovine serum albumin, gelatin, and lysozyme dramatically reduced the immunotoxicity of nanocomposites^31^. From the other hand the possibility of Fe(0) @ MCM-41 to activate ROS-mediated signaling pathways could be exploited in cancer treatment. Thus, upon irradiation of tumor cells that incorporated SeC @ MSNs-Tf/TAT nanoparticles, the latter induced overproduction of ROS inside cells, which activated the mechanisms of apoptosis by activating p53, AKT and MAPKs pathways^15^. Additional incorporation of the ROS-generating drugs can potentially increase the therapeutic potency of MSNs. In the study by Huang *et al*. it was shown that micelles of SPIONs which included β-lapachone (β-lap) known to selectively increase ROS production have a significant cytotoxic effect^33^.

Administration of the Fe(0) @ MCM-41 via intravenous injection accompanied by the incorporation of the nanoparticles in the tumor (Fig. 5). Thus we observed a significant retention of the MSNs in the perivascular spaces in the glioma on the cryosections of the tumor (Fig. 5B). This led to the enhancement of the tumor contrast on gradient echo MR images obtained by the T2-weighted scans (Fig. 5A). The MR pattern of MSNs presentation in the tumor was similar to the earlier reported non-specific accumulation of the SPIONs in the C6 glioma^34^,^35^,^36^.

**Figure 6:**
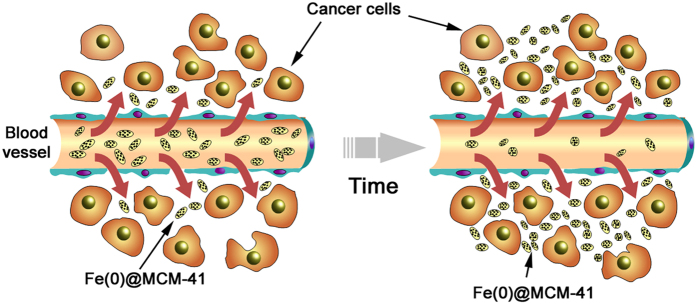


The observed accumulation of the Fe(0) @ MCM-41 nanoparticles in the glioma could be explained by the increased blood-brain barrier permeability in the tumor^18^. Previously it was found that glioma tumors disrupt the connection of astrocytes and brain endothelial cells^37^. Thus in the study Watkins *et al*. it was shown that invading along the preexisting blood vessels glioma cells disrupt the astrocytic end-feet attachment from endothelial cells^38^. Moreover, decreased expression of the tight junction proteins (i.e., occludin) as well as overexpression of aquaporin-4 result in the impairment of the endothelial tight junctions^31^,^39^. The abnormalities of tumor tissue architecture and vasculature leakage could also be responsible for the phenomenon of enhanced permeability and retention (EPR) effect^40^. This effect provides the basis for selective delivery of macromolecular drugs to the site of the solid tumors. Thus Fe(0) @ MCM-41 achieve very high local intratumoral concentrations within 24 hours after administration (Fig. 6).

One of the conclusions from the obtained data is the possibility of Fe(0) @ MCM-41 to reach the brain tumor. The porous structure of MSNs could be applied for the prolonged drug delivery. Thus in the work of Gordon *et al*. the possibility of prolonged drug dissolution (e.g., acetaminophen, progesterone, stavudine) by MSNs of various formulations was shown^41^. In another study formulated multifunctional inorganic mesoporous nanocapsules (Fe(3)O(4) @ mSiO(2)) demonstrated high efficiency in doxorubicin release^42^. Intra-tumorally accumulated MSNs could be also applied for the hyperthermia of the brain tumors in alternating magnetic field that might be further enhanced with radio- or radioimmunotherapy^43^,^44^. Thus as was shown in phase III trials combination of radiotherapy and hyperthermia in comparison to the radiotherapy alone proved to be beneficial in terms of local control and survival^45^,^46^.

The obtained results suggest that MSNs are suitable for penetrating the blood-brain barrier of tumors.

## Materials and Methods

### Synthesis and characterization of Fe(0) @ MCM-41 nanoparticles

For the synthesis of mesoporous silica particles the surfactant CTABr (cetyltrimethylammonium bromide) was dissolved in a mixture of water/ethanol/ammonia and then tetraethylorthosilicate (TEOS) was added to this solution as a precursor of the silica as described earlier^47^,^48^. The reaction mixture was stirred for 2 hours for aging of the silica gel. The obtained precipitate was dried in the air at 473 K for 1 hour and after was calcinated at temperature 873 K (heating rate was 5 K/min) for 40 minutes.

The metal-containing MSNs derivatives were synthesized by insertion of ferric chloride (III) solution (50% from saturated concentration) in the porous of the silica. The Fe metal precursor was added to the calcinated pre-formed MSNs by vacuum impregnation at residual pressure of 0.1 mm Hg followed by removing the ferric chloride excess from the surface of the silica grains by washing with methylene chloride. For the reduction of FeCl_3_ to Fe^0^ samples were put in the H_2_ flow at 600 °C for 6 hours.

The morphology of the obtained Fe(0) @ MCM-41 nanoparticles was controlled by scanning electron microscopy (JEOL JEM 3010). The field-emission scanning electron microscopy (FE-SEM Hitachi SU8020) equipped with an energy disperse X-ray spectrometer was employed for element analysis. The structural properties of MSNs impregnated with zero-valent Fe were analyzed by means of Bruker D2 Phaser diffractometer (XRD) supplied with a Cu sealed tube (X-ray source; λ = 0.1542 nm) and a LYNXEYE detector and as well as by a standard Mössbauer spectrometer (POLON) provided with a ^57^Co/Rh source of γ-radiation. Magnetic properties of Fe(0) @ MCM-41 nanoparticles were studied using a magnetometer (Oxford Instruments Ltd). Additionally the magnetic properties of conjugates were verified by measurements of second harmonic generation by them in parallel weak *ac* and steady state magnetic fields (NLR-*M*_*2*_).

### Analysis of MSNs incorporation into normal and tumor cells

#### Cells

All the cell lines: (i) rat C6 glioma; (ii) HeLa; (iii) U87; and (iv) K562 were kindly provided by the Institute of Cytology of the Russian Academy of Sciences (St. Petersburg, Russia) from the Russian Cell Culture Collection. HeLa as well as C6 cells were cultured in CO_2_-incubator (at 37 °C in presence of 6% CO_2_) in DMEM supplemented by 2 mM L-glutamine, 10% fetal bovine serum (FBS), and antibiotics: Streptomycin (100 units/mL) and Penicillin G (100 μg/mL). U87 and K562 cells were cultured in complete RPMI-1640 cell medium augmented by 10% FBS.

#### Peripheral blood mononuclear cells (PBMCs)

Rat monocytes were isolated from whole blood using ficoll gradient centrifugation. Cells were harvested in RPMI-1640 supplemented by 2 mM L-glutamine, 10% FBS as well as antibiotics: Streptomycin (100 units/mL) and Penicillin G (100 μg/mL).

#### Fibroblasts

Rats’ dermal fibroblasts were obtained from cells that migrated from rabbit skin fragments as was described earlier^49^. Briefly, the skin fragments were cut into small, 0.1 × 0.1 cm pieces that were cultured in DMEM cell medium, augmented by 2 mM L-glutamine, 10% fetal bovine serum (FBS) as well as antibiotics (0.1 mg/ml streptomycin and 100 U/ml penicillin G). In 3–4 weeks, a confluent fibroblast cell monolayer was developed on a plate surface. The cell viability was determined by 0.4% trypan blue exclusion.

#### Analysis of cytotoxicity

For analysis of nanoparticles cytotoxicity the non-radioactive cytotoxicity assay CytoTox 96 (Promega Inc., USA) was applied in accordance with the manufacturer’s protocol. Briefly, C6 glioma, U87, HeLa cells, K562, PBMCs and fibroblasts were co-incubated during 1, 3, 12 and 24 hours with MSNs or Fe(0) @ MCM-41 taken at different concentrations (1, 5, 10, 50, and 150 μg/ml). Then supernatants were collected and concentration of lactate dehydrogenase (LDH) in the samples was determined.

#### Determination of ROS

Production of ROS in tumor and normal cells was detected using a dichlorodihydrofluorescein diacetate (DCDHF) fluorescent probe (C2938) obtained from Molecular Probes. Cells were exposed to 10 μM DCDHF in serum-free medium for 30 min at 37 °C. Then cells were washed and incubated in phenol-red-free medium with MSNs and Fe(0) @ MCM-41 particles for 1, 3, 12 and 24 hours. Fluorescence monitoring was fulfilled by employment of a microplate fluoremeter with wavelengths of 485 (for excitation) and 538 nm (for emission).

#### Confocal microscopy of nanoparticles

The confocal microscopy was applied for assessment of cellular interactions of nanoparticles. C6 glioma, HeLa cells, U87, K562, PBMCs and fibroblasts (1 × 10^6^ cells/ml) were permitted to settle on glass slides coated by poly-L-lysine. Cells were co-incubated for 1, 3, 12 and 24 hours with MSNs or Fe-MSNs (both at 150 μg/ml) in CO_2_-incubator at 37 °C. Nuclei staining with 4,6-Diamidine-2-phenylindole solution (DAPI) (Vector Laboratories, Burlingame, CA, USA) was used for all types of cells. Fluorescence images were registered with using a Leica TCS SP8 confocal technique (Leica Microsystems, Heidelberg, Germany).

#### Transmission electron microscopy

MSNs or Fe(0) @ MCM-41 particles were co-incubated with cells for 24 h cells at concentration of 150 μg/ml. Following incubation cells were fixed for 1 h at 4 °C in 2.5% glutaraldehyde dissolved in 0.1 M cacodylate buffer with pH 7.4. To adapt the samples for electron microscopy the cells were embedded in Epon and Araldit, and then sectioned in a LKB ultratome by a diamond knife. Utrathin sections placed on fine mesh copper or nickel grids, were finally colored with uranyl acetate and lead citrate for investigation by electron microscope (Zeiss Libra 120).

### Animal experiments

#### Animals

Wistar rats (male) with weight 300–320 g were obtained from animal nursery “Rappolovo” RAMN (St. Petersburg, Russia). The approvement have been provided by the local ethical committee of I.P. Pavlov State Medical University (St. Petersburg, Russia) for all experiments with animals in accordance with institutional guidelines for the welfare of animals.

#### C6 glioma orthotopic model

Rodents were anesthetized intraperitoneally with 0.2 ml 2% Rometar (Bioveta, Czech Republic) and 10 mg “Zoletyl-100” (Vibrac sante Animale, France) before they were positioned into the stereotactic frame (David Kopf Instruments, Tujunda, CA). C6 glioblastoma cells (5 × 10^6^ cells/ml) in 15 μl were used for injection into the *nucl.caudatus dexter*.

#### Brain tumor imaging

On the 15^th^ day after inoculation for comparative analysis of the magnetic nanoparticles retention in the tumor, rodents were randomly divided into four groups (6 animals each) with purpose of: (1) the phosphate-buffer solution (PBS) injection by i.v. (control group); (2) i.v. injection of MCM-41; (3) i.v. injection of the Fe(0) @ MCM-41 (2.5 mg/kg) for 24 hours; (4) i.v. injection of Fe(0) @ MCM-41 (10 mg/kg) for 24 hours. Brain tumors were imaged using high-field (11.0 T) MRI scanner (Bruker). T_2_-weighted images (TR 4200 ms; TE 36 ms and Flip angle 180°) were obtained in coronal plane with slice thikness 1.0 mm. Additionally we performed T_1_-weighted images (TR 1500 ms; TE 7.5 ms and Flip angle 180°) as well as gradient echo images (TR 350 ms; TE 5.4 ms and Flip angle 40°) in coronal planes. Retention of the Fe(0) @ MCM-41 particles in the glioma was assessed using multi-slice multi-echo (MSME) sequences. Analysis of images was carried out with application of software Analyze (AnalyzeDirect, Inc., Overland Park, KS). For estimation of a change of contrast on the T_2_ maps the calculations of T_2_ values were fulfilled with the help of software package Paravision 3.1 (Bruker BioSpin GmbH, Rheinstetten, Germany).

#### Histological analysis

Animals were sacrificed using 150–200 mg/kg of pentobarbital (administered intra-peritonealy) and then perfused with 100 ml of saline and 4% paraformaldehyde for histological analysis. The whole brains were collected and fixed in 4% paraformaldehyde and 30% sucrose. The embedded in Tissue-Tek compound blocks were cut into serial 5–7 μm sections for histology. Sections were treated additionally by DAPI. Fluorescence data were acquired by confocal technique Leica TCS SP8 (Leica Microsystems, Heidelberg, Germany). Reflecting laser scanning with laser exciting at 488 nm (Ar/Kr) was applied for detection of Fe(0) @ MCM-41 particles.

### Assessment of Fe(0) @ MCM-41 biodistribution

Measurements of nonlinear longitudinal response (NLR-*M*_*2*_) under action of *dc* and *ac* magnetic fields with parallel orientation, *H* + *h*·sin(2*πft*) (*h* = 13.8 Oe, *f* *=* 15.7 MHz), were applied for analysis of the brain tumor targeting efficiency by mesoporous silica nanoparticles and biodsitribition assessment. The symmetric relative to the point *H* = 0 and periodic with frequency *F*_sc_ = 8 Hz scanning of *dc* field *H* was employed in all the experiments except of experiment *in vitro* with solution of Fe(0) @ MCM-41 NPs (similar to that used for injections), where the value of *F*_sc_ was changed in the range 8 ÷ 10^−2^ Hz to clarify the magnetic state of NPs. The symmetrical *H-*scan was convenient for observation of signal field hysteresis.

In 24 hours after intravenous infusion of nanoparticles (or PBS in control group) rodents were sacrificed and tissue’s probes (mass of every one being approximately 100 mg) were taken from organs and tumor (3 samples from each organ and glioma) for magnetic measurements. There were probes from heart, spleen, kidney, lungs, liver, skin, muscle as well as from normal brain and glioma. Both phase components Re*M*_2_ and Im*M*_2_ of second harmonic of nonlinear response were registered as function of steady field *H*. The nonlinearity of response was provided by a presence of magnetic nanoparticles in samples under study, their signal being recorded against a background signal of molecules in possible paramagnetic state in tissues^50^. Adapted for studying of magnetic nanoparticles, described earlier, home-made installation was employed in this study^51^,^52^. The integral sensitivity of *M*_2_- measurements was 10^−10^ emu^52^.

### Statistics

The one-way analysis by Kruskal-Wallis was applied for detection of the differences. Comparisons of data for the groups of rodents under study were assessed using Student’s *t-*test. The software program used for the statistical analysis was Statistica Version 9.2. In all experiments, distinctions were regarded as statistically reliable at *P* < 0.05 53.

## Additional Information

**How to cite this article**: Shevtsov, M. A. *et al*. Zero-valent Fe confined mesoporous silica nanocarriers (Fe(0) @ MCM-41) for targeting experimental orthotopic glioma in rats. *Sci. Rep.* **6**, 29247; doi: 10.1038/srep29247 (2016).

## Supplementary Material

Supplementary Information

## Figures and Tables

**Table 1:** ReM2(H) parameter for tissue samples from animals treated with PBS, Fe(0) @ MCM41 at 2.5 mg or 10 mg.

Tissue sample	Control (10^−8^ emu/g)	Fe(0) @ MCM-41 (2.5 mg) (10^−8^ emu/g)	Fe(0) @ MCM-41 (10 mg) (10^−8^ emu/g)	A_10 mg_/A_2.5 mg_
brain	0.034(22)	0.20(2)	0.13(2)	0.65(18)
tumor	0.074(22)	0.083(22)	63.88(2)	770(204)
liver	0.043(22)	30	80.20(2)	2.67(1)
lungs	0.044(22)	0.468(22)	25.20(2)	54(3)
kidney	0.020(22)	0.70(2)	0.131(22)	0.19(4)
spleen	0.029(22)	58.07(2)	71.61(2)	1.23(1)
heart	0.454(22)	0.132(22)	0.132(22)	1.0(2)
skin	0.140(22)	0.57(2)	0.461(22)	0.81(7)
muscle	0.046(22)	0.11(2)	0.121(22)	1.1(4)

Amplitudes of signals with H-hysteresis (including control signals) were calculated as: {|ReM_2ext|for direct *H*-scan at *H*<0_ + |Re*M*_2ext_|_for reverse *H*-scan at *H*>0_}/2 since extremum can be absent at *H* > 0 for direct *H*-scan and at *H* < 0 for reverse *H*-scan. Amplitudes of signals with a weak *H*-hysteresis were calculated as: {|Re*M*_2ext_|_for direct *H*-scan at *H*<0_ + |Re*M*_2ext_|_for direct *H*-scan at *H*>0_}/2.
