# Supplementary material for: Zero-valent Fe confined mesoporous silica nanocarriers (Fe(0) @ MCM-41) for targeting experimental orthotopic glioma in rats
Source: Sci Rep. 2016 Jul 8;6:29247. doi: 10.1038/srep29247 (PMC4937429; doi:10.1038/srep29247)
Supplement: Supplementary Information [file srep29247-s1.pdf]

Supplementary information

Zero-valent Fe confined mesoporous silica nanocarriers (Fe(0)@MCM-41) for targeting experimental orthotopic glioma in rats

Shevtsov M.A., Parr M.A., Ryzhov V.A., Zemtsova E.G., Arbenin A.Yu., Ponomareva A.N., Smirnov V.M., Multhoff G.

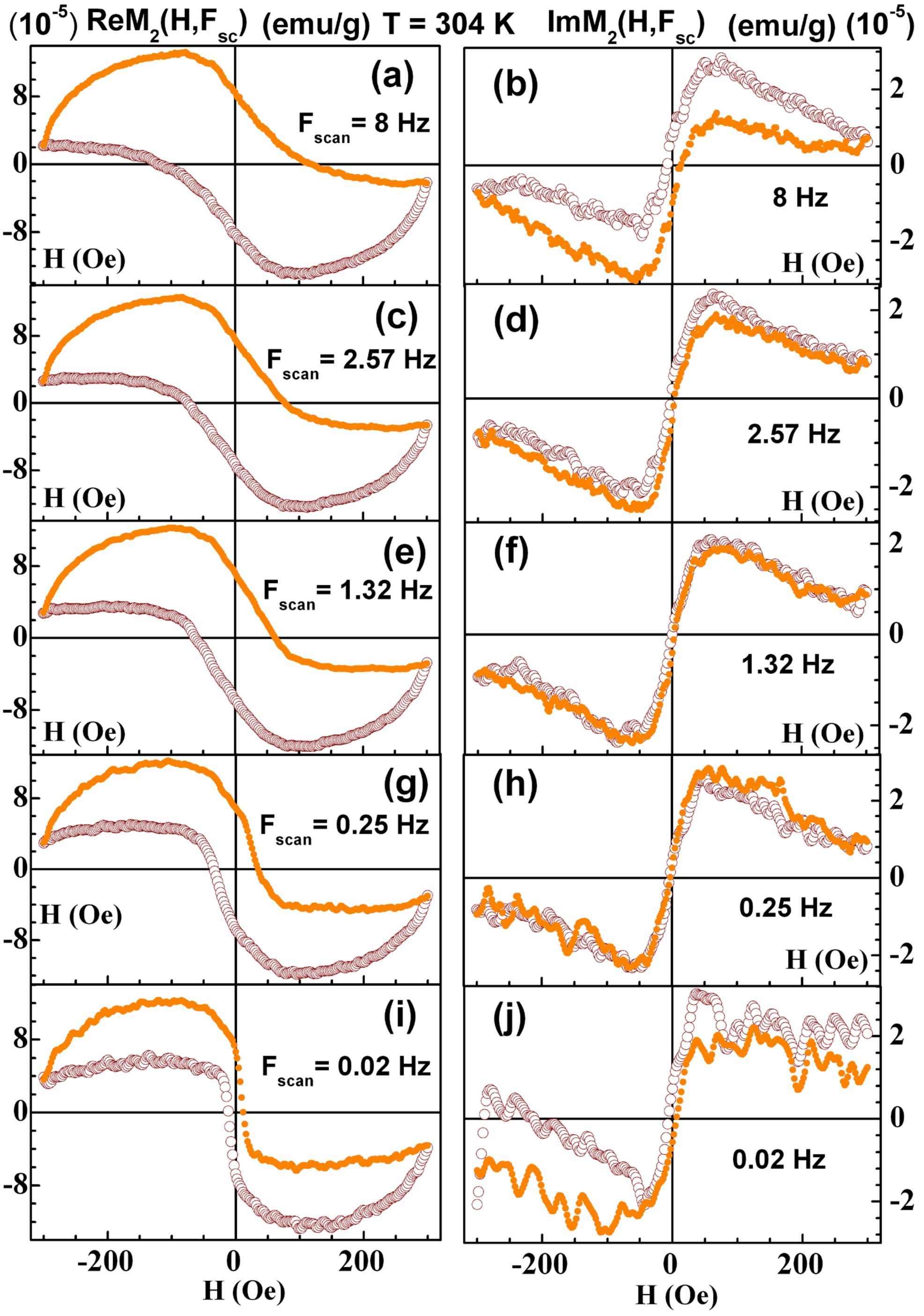

Supplementary Figure S1. The phase components, ReM_2 and ImM_2 were registered simultaneously as functions of steady field H at T = 294 K for various values of field scan, F_{sc} under condition $M_2 \propto h^2$ (h – is amplitude of *ac* field).

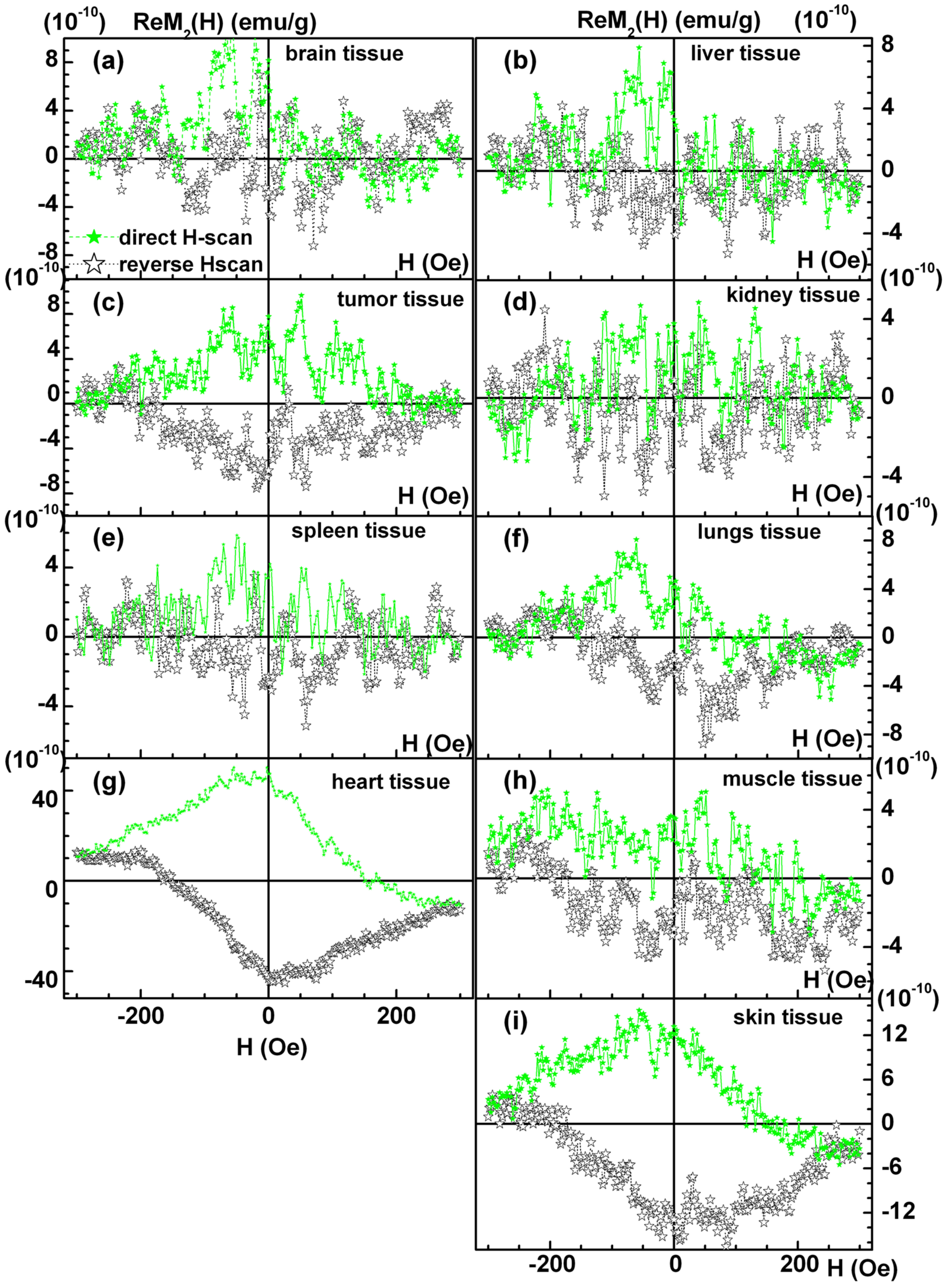

Supplementary Figure S2. **Re$M_2(H)$ part of the second harmonic of magnetization from control tissues.**

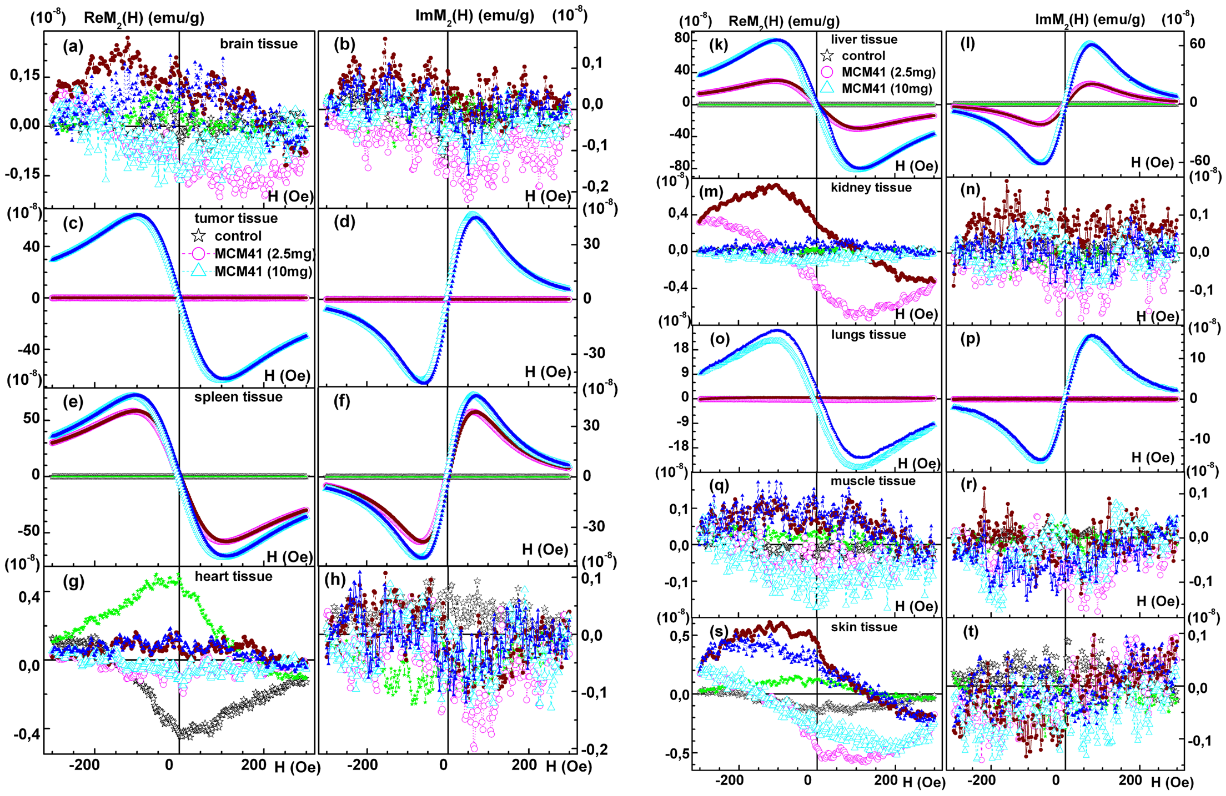

Supplementary Figure S3. Biodistribution of Fe(0)@MCM-41 nanoparticles in animals characterized by NLR-*M_2* measurements. Second harmonic measurement for brain, tumor, spleen, heart, liver, kidney, lungs, muscle and skin tissues.
